# Comparison of Nutritive Values of Tropical Pasture Species Grown in Different Environments, and Implications for Livestock Methane Production: A Meta-Analysis

**DOI:** 10.3390/ani12141806

**Published:** 2022-07-14

**Authors:** Priyanath Jayasinghe, Thiagarajah Ramilan, Daniel J. Donaghy, Keith G. Pembleton, David G. Barber

**Affiliations:** 1 School of Agriculture and Environment, Massey University, Private Bag 11 222, Palmerston North 4440, New Zealand; t.ramilan@massey.ac.nz (T.R.); d.j.donaghy@massey.ac.nz (D.J.D.); 2 Department of Animal Science, Faculty of Animal Science and Export Agriculture, Uva Wellassa University, Badulla 90000, Sri Lanka; 3 Centre for Sustainable Agricultural Systems and School of Agriculture and Environmental Science, University of Southern Queensland, Toowoomba, QLD 4350, Australia; keith.pembleton@usq.edu.au; 4 Agri-Science Queensland, Department of Agriculture and Fisheries Queensland, University of Queensland Gatton Campus, Lawes, QLD 4343, Australia; david.barber@daf.qld.gov.au

**Keywords:** dairying, grasses, methane, meta-analysis, tropical pasture, quality

## Abstract

**Simple Summary:**

Globally, tropical pasture species predominate in tropical and subtropical climates, and are the primary feed source for grazing livestock including dairy cattle. Therefore, this study aimed at systematically analysing the nutritive values of tropical pastures and the implications of potential methane gas production from tropical pasture species and livestock, in relation to the growing conditions and agronomic management approaches used (defoliation frequency and intensity) across different climates. This analysis allowed us to understand the better performing tropical pasture species grown across wider geographical regions, in order to improve pasture-based livestock production systems. Results revealed that pasture quality and methane gas production varied among and within species, and were significantly affected by the climate and by the agronomic management regime as well.

**Abstract:**

The demand for dairy products is ever increasing across the world. The livestock sector is a significant source of greenhouse gas (GHG) emissions globally. The availability of high-quality pasture is a key requirement to increase the productivity of dairy cows as well as manage enteric methane emissions. Warm-season perennial grasses are the dominant forages in tropical and subtropical regions, and thus exploring their nutritive characteristics is imperative in the effort to improve dairy productivity. Therefore, we have collated a database containing a total of 4750 records, with 1277 measurements of nutritive values representing 56 tropical pasture species and hybrid cultivars grown in 26 different locations in 16 countries; this was done in order to compare the nutritive values and GHG production across different forage species, climatic zones, and defoliation management regimes. Average edaphoclimatic (with minimum and maximum values) conditions for tropical pasture species growing environments were characterized as 22.5 °C temperature (range 17.5–29.30 °C), 1253.9 mm rainfall (range 104.5–3390.0 mm), 582.6 m elevation (range 15–2393 m), and a soil pH of 5.6 (range 4.6–7.0). The data revealed spatial variability in nutritive metrics across bioclimatic zones and between and within species. The ranges of these nutrients were as follows: neutral detergent fibre (NDF) 50.9–79.8%, acid detergent fibre (ADF) 24.7–57.4%, crude protein (CP) 2.1–21.1%, dry matter (DM) digestibility 30.2–70.1%, metabolisable energy (ME)3.4–9.7 MJ kg^−1^ DM, with methane (CH_4_) production at 132.9–133.3 g animal^−1^ day^−1^. The arid/dry zone recorded the highest DM yield, with decreased CP and high fibre components and minerals. Furthermore, the data revealed that climate, defoliation frequency and intensity, in addition to their interactions, have a significant effect on tropical pasture nutritive values and CH_4_ production. Overall, hybrid and newer tropical cultivars performed well across different climates, with small variations in herbage quality. The current study revealed important factors that affect pasture nutritive values and CH_4_ emissions, with the potential for improving tropical forage through the selection and management of pasture species.

## 1. Introduction

Demand for dairy products is ever increasing across the world; however, the livestock sector is a significant source of global greenhouse gas (GHG) emissions [1]. Forage plants provide feed for an estimated 1.5 billion cattle and 0.2 billion buffalo around the world, supplying humans with our daily protein requirements [2]. Approximately 47% of global milk comes from tropical and subtropical regions [3]. Due to increasing populations in developing countries, global food production needs to substantially increase in the future; the demand is expected to nearly double by 2050 [4]. Parallel to the expansion of food production, GHG emissions have climbed; anthropogenic emissions account for a substantial proportion (58%) of these global emissions [5], with 18% (5.0–5.8 Gt CO_2_eq yr^−1^) of that generated by agriculture [6], contributing 25% of methane (CH_4_) production from the enteric fermentation of ruminants [4]. Dairy production in tropical regions is often lower than in temperate regions, due to a range of factors including lower nutritive value of forage grasses, lack of access to inorganic nitrogen (N) fertiliser, infertile soils, and adverse climatic conditions [7]. Mostly tropical cattle are fed a diet that is based essentially on pasture species that generally have low nutritive value, with large seasonal variations in quantity and quality [8]. However, nutrient intensification through planting improved forage options [9], in addition to adopting appropriate harvesting, fertilizing, and grazing practices [10], have substantially improved the nutritive value of tropical pastures.

The largest differences in nutritive values of herbage have been observed among different groups of forages (grasses and legumes, tropical and temperate species), but there are still substantial differences between species and even between different cultivars within a species [11]. Improving dairy cattle nutrition through adequate feeding of quality forage, choice of appropriate herbage species and cultivars depends on the adaptability of selected species to the farm environment and the resulting balance between quantity and quality [11]. However, a paucity of data that compares the nutritive values of tropical pastures grown across different environments limits the selection of forages for livestock. Therefore, gauging the performances of different pasture species and cultivars across different environments aids in identifying different spatial and temporal niches that are suitable for growing tropical pastures, in order to improve dairy cattle nutrition and lower GHG emissions in the tropics.

The age of the plants at their time of utilisation (by grazing or cutting) is an important aspect to consider in tropical pasture production, as this significantly affects their yield and chemical composition, which consequently affects milk production [12]. While dry matter (DM) production significantly increases with maturity, the digestibility and the crude protein (CP) content both rapidly decline with advancing plant maturity [13,14]. Although forage maturity reduces its nutritive value, environmental and agronomic management factors can alter the relationship between plant maturity and forage nutritive value [13,15]. Herbage accumulation and the nutritive value of grasses are both strongly affected by management factors, such as defoliation (grazing and cutting) frequency, intensity, and the application of N fertiliser (amount and frequency) [16,17]. Sward characteristics are highly sensitive to environmental conditions such as rainfall, air temperature, and incident solar radiation [18]. Therefore, herbage nutritive value might vary considerably in plants harvested at the same stage of maturity, if they are grown in different environments. Variable environmental conditions (e.g., increased temperature, low precipitation) can induce physiological and phenological changes in plants to delay or to hasten anthesis, which is associated with greater stem production (increased stem:leaf ratio) [19,20], leading to variations in nutritive value. Therefore, systematically assessing the nutritive values of various tropical pasture species in relation to the growing conditions and agronomic management approaches (defoliation frequency and intensity) across different climates allows some of these interactions to be identified; in turn, this information can provide a basis to explore options for intensification of tropical livestock production systems.

In addition to increasing milk production, improving the nutritive value of tropical pasture species may also have environmental co-benefits in terms of reducing GHG emissions [10]. Forages generally have enhanced nutritive value for livestock if they contain a greater proportion of readily fermentable components, such as sugars, organic acids, and proteins, as well as a lower proportion of fibre [21]. The chemical composition and morphology of pasture species determines their preference and subsequently influences the feed intake, the efficiency of rumination, the rates of weight gain, the quality and volume of milk produced, and the reproductive performance of grazing ruminants [22]; moreover, the chemical composition and morphology of pasture species may also change the environmental footprint of livestock products in terms of CH_4_ emissions. Increased pasture nutritive value can reduce CH_4_ emissions by 5% with increasing total tract neutral detergent fibre digestibility (NDFD) [23], and this also is expected to increase the production of volatile fatty acids (VFA), thereby increasing milk production [23]. Furthermore, better nutritive values of herbages increase the DM intake (DMI) and subsequently decrease CH_4_ emissions by 2–6% for each kilogram of increased DMI [23].

Despite the array of individual studies conducted that relate to tropical pasture production and nutritive value assessment, no study has accessed the multiple nutritive metrics of commonly cultivated tropical pasture species and newer cultivars in different agroclimatic conditions under varying defoliation management regimes (frequency and intensity). Further, subsequent effects of nutritive value on possible CH_4_ emissions have not been well elucidated. Therefore, this meta-analysis aimed at the following: (i) to compare the nutritive value variation between tropical pasture species grown in different bioclimatic environments with implications for subsequent CH_4_ production from dairy cows; and (ii) to evaluate the effect of defoliation interval, intensity, and bioclimate on nutritive values and CH_4_ emissions using a georeferenced tropical pasture nutritive metrics database.

## 2. Materials and Methods

### 2.1. Data Collection

We constructed a georeferenced database from the tropical forage experimental data. Experiments that were included were identified by systematically searching the available literature on the Discover, Web of Science, Scopus, and Google Scholar databases from 15 March–20 April 2021. Initial search terms used were ‘defoliation’, ‘harvesting time’, ‘cutting interval’, ‘methane gas’, ‘CH_4_ gas’, ‘emission’, combined with ‘tropical pasture’, ‘pasture quality’, and ‘nutritive value’. The searched articles were included in the database if the nutritive value or chemical composition had been analysed for a specific tropical grass species, or for those that were hybrid grown and tested under field conditions at a defined location and harvested more than once. This meta-analysis did not intend to explore the tropical legumes, herbs, and cereal grasses; therefore, such articles were excluded. All experiments that tested in climate-controlled environments (e.g., glasshouses) were also excluded from the database.

Collated articles were closely scrutinized based on important descriptive statistics, in order to be considered as data input for the analysis. Experimental, site-specific geospatial data (latitude, longitude, altitude, mean sea level (MSL)), as well as climate parameters (mean annual temperature (MAT) and mean annual rainfall (MAR)) were also recorded where available. Google Earth Pro Version 7.3.1 (Google Inc, Mountain View, CA, USA) was used to supplement missing geospatial data for certain locations. Similarly, missing climate variables were obtained from NASA POWER data access viewer (https://power.larc.nasa.gov (accessed on 22 April 2021) by generating agroclimatology files. Site-specific climate data were used with criteria in the Köppen-Geiger climate classification [24], in order to identify the climatic types for each experimental location. Soil physical properties such as soil type and soil pH were also included, where possible, in order to describe the experimental site. All species and hybrids were designated with unique codes; in addition, for each grass species, defoliation frequency and nutritive value metrics were assigned to a new row as a treatment in the database. Defoliation frequency was recorded in days for each defoliation event. In total, 35 individual studies consisting of 294 treatments were included in the database (Supplementary Table S2).

Dry matter yield of the pasture species was recorded in units of t ha^−1^ year^−1^. Organic matter (OM) and forage nutritive values (CP, acid detergent fibre (ADF), NDF, and acid detergent lignin (ADL)) were included if analysed by either the Association of Official Agricultural Chemists (AOAC, 1975) or by near-infrared spectroscopy (NIRS) methods and recorded as either percentages of DM or g kg^−1^. Wherever forage CP was analysed using wet chemistry and expressed as N content, CP was estimated by multiplying the determined nitrogen (N) content by 6.25. Tropical forage digestibility metrics (DM digestibility (DMD) and OM digestibility (OMD)) were included in the database if they were estimated using in vivo, in vitro or NIRS techniques, and if forage energy values (MJ kg^−1^ DM) were expressed in terms of metabolisable energy (ME) [25].

### 2.2. Data Processing and Analyses

The metabolisable energy [25] and OMD [26] of each tropical pasture were estimated using the following equations:

ME = (0.16 × OMD) − 1.8(1)

OMD (%) = 3.802 + 0.97051 × DMD (%)(2)

Methane gas (g CH_4_ animal^−1^ day^−1^) was estimated using the NDF and DMI in the database for tropical pasture species using a published relationship. This selected equation that follows was a product of a meta-analysis [26] where the database consisted mainly of tropical pastures fed to cattle in tropical regions:

CH_4_ (g CH_4_ animal^−1^ day^−1^) = 17.0 (±0.99) × DMI + 0.03 (±0.01) × NDF (3)

According to the meta-analysis of Lee et al. [27], the average DMI of tropical cattle was assumed to be 7.7 kg day^−1^ [28].

Nutritive value parameters and CH_4_ production were descriptively analysed and correlated with frequency of defoliation.

Following data processing, weighted, restricted maximum likelihood linear mixed-effects (LME) models were fitted [29], as shown below, in order to determine the nutritive value and CH_4_ production variation between bioclimatic zones as well as the effects of environmental and management determinants:

(4) $$Y_{ij}=B_{0}+b_{0}+B_{1}\times 1ij+b_{1}\times {\;}_{1ij}+B_{2}\;\times_{\;2ij}+......+B_{p}\;X_{pij}+e_{ij}$$

where $Y_{ij}$ represents the nutritive value parameters and CH_4_ in the *i^th^* treatment of the *j^th^* study; $B_{0}$, $B_{1}\times_{\;1ij},\;B_{2}\;\times_{\;2ij}$…., $B_{p}\;X_{pij}$ are the fixed effects (intercept and effects of independent variables); $b_{0}$ (intercept) and $b_{1}$(slope) are the random study effects (***i*** = 1…. n treatments and ***j*** = 1…. n studies); and $e_{ij}$ is the sampling error.

Model fitting was carried out by including nutritive metrics and CH_4_ as response variables with multiple potential explanatory variables and their interactions. Defoliation frequency and defoliation intensity, along with the climatic type, were added as fixed effects. Different cultivars from the same site were recorded in the database and were repeatedly measured for the same nutritive metrics; therefore, when defining random effect, observations (treatment) were nested within cultivars, and different cultivars in the same study were nested within the study (site) in the LME model. Furthermore, mean annual rainfall, mean annual temperature, and mean sea level were added as covariates. Soil pH and soil type were shown to have no significant relationships (*p* > 0.05) with nutritive metrics and CH_4_; thus, these variables were not considered in the initial LME model. All the data were analysed using R statistical computing software (R Foundation for Statistical Computing, Vienna, Austria) [30].

## 3. Results

### 3.1. Description of the Analytical Database

**Figure 1:**
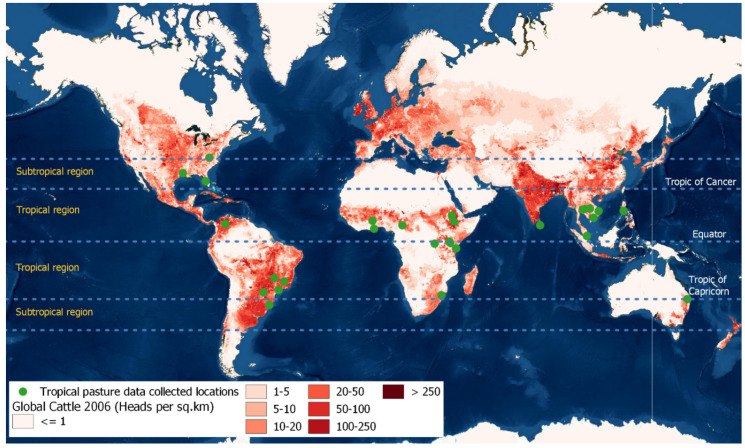
Spatial distribution of the tropical pasture data. Base map: global cattle distribution using the Gridded Livestock of the World 2 global distribution [31] derived from ArcGIS Pro 2.8.6 (ESRI, California, USA) [32].

The database contained a total of 4750 records, with 1277 measurements of nutritive value parameters representing 56 tropical pasture species and hybrid cultivars grown in 26 different tropical environments in 16 countries (Figure 1). In some studies, not all of the chosen variables were reported; therefore, the number of observations was not uniform (Table 1). According to the collated data, tropical forage growing environments were characterized as 22.5 °C MAT (range 17.5–29.30 °C), 1253.9 mm MAR (range 104.5–3390.0 mm), 582.6 m MSL (range 15–2393 m), and 5.6 (range 4.6–7.0) mean soil pH.

Crude protein was the most commonly measured nutritive value (in 83% of the records), followed by NDF (70%), and ADF (67%). Acid detergent lignin was the least measured metric (15%), followed by OM (19%). According to the database of the present study, records were the most numerous from the tropical/equatorial bioclimatic zone, comprising 55% of the data set, compared with 36% from the warm temperate and 9% from the arid/dry bioclimatic zones. Multiple nutritive metrics contained in tropical/equatorial bioclimatic zone studies contributed to the largest total number of measurements in the database. Interestingly, genus *Brachiaria* was the commonly reported (34%) tropical pasture, followed by *Cynodon* (19%), and *Pennisetum* (16%). Within the genus, *Brachiaria Mulato II* was the most popular cultivar (20%). Descriptive statistics of key variables across all types of tropical pasture species are summarised in Table 1. There were large differences between minimum and maximum values (see the Table 1) in the data set for climate (MAT, MAR, MSL), defoliation frequency (days), DM yield, and nutritive values (CP, ADF, DMD, OMD, and ME).

### 3.2. Comparisons of the Nutritive Values

**Figure 2:**
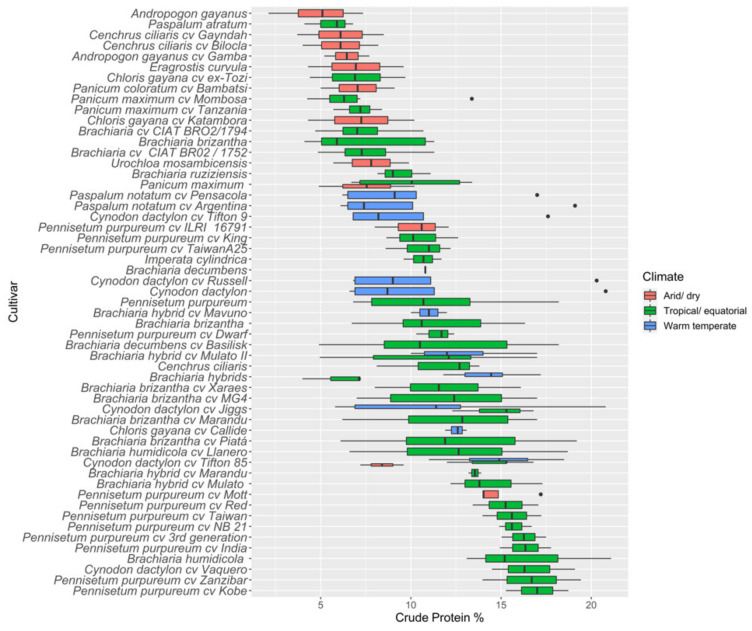
Ascending median values of crude protein (% DM) in tropical pasture species and cultivars grown in different environments.

**Figure 3:**
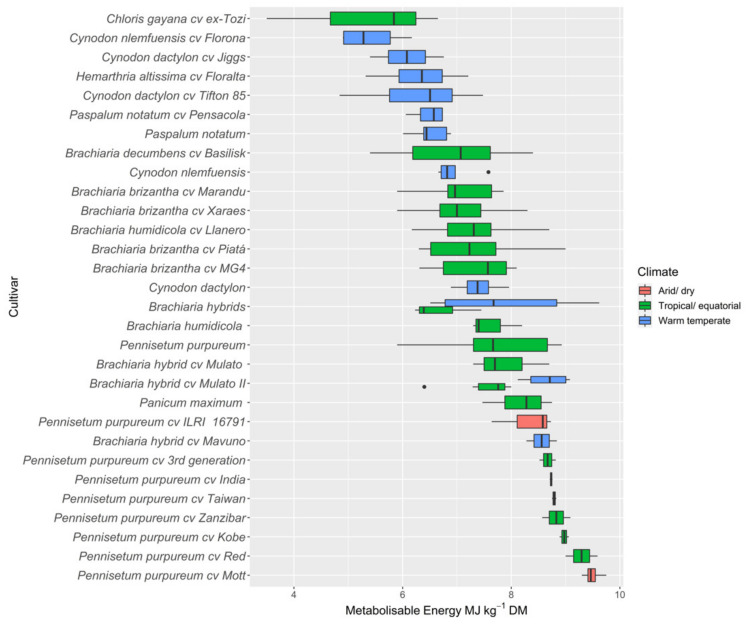
Ascending median values of metabolisable energy (MJ kg^−1^ DM) in tropical pasture species and cultivars grown in contrasting environments.

**Figure 4:**
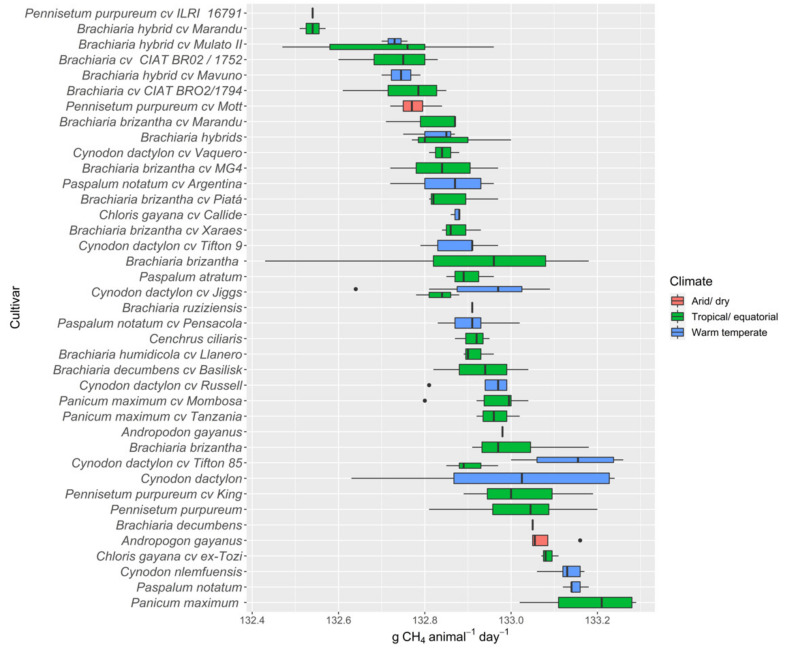
Ascending median values of estimated methane production (g CH_4_ animal^−1^ day^−1^) in tropical pasture species and cultivars grown in contrasting environments. Methane gas production was estimated using CH_4_ (g CH_4_ animal^−1^ day^−1^) = 17.0 (±0.99) × dry matter intake + 0.03 (±0.01) × neutral detergent fibre.

Nutritive values across all tropical pasture species are summarised (Supplementary Table S1), and ascending median values of main nutritive value components (CP and ME) and CH_4_ production are presented in Figure 2, Figure 3 and Figure 4, respectively. There was substantial variations in nutritive values both within and between species in different environments.

#### 3.2.1. Crude Protein

The largest absolute CP values were recorded from the grasses *Pennisetum purpureum cv Kobe* at 17%, *Pennisetum purpureum cv Zanzibar* at 16.7%, and *Brachiaria humidicola* at 16.4%. Minimum average CP values were measured for *Andropogon gayanus* at 4.6%, *Chloris gayana cv ex-Tozi* at 7%, and *Panicum maximum cv Mombosa* at 7.1%, across all growing environments. Among all tropical pasture species, *Pennisetum* species reported greater mean CP percentages, followed by *Brachiaria*; in contrast, *Paspalum* and *Panicum* species had lower CP values. Between species, *Brachiaria* (7.36–16.4%) and *Pennisetum* (10.2–17%) recorded the largest ranges of CP values; within species, *Brachiaria* had higher variations.

#### 3.2.2. Fibre

Fibre components of the tropical pasture species were recorded in terms of NDF and ADF percentages. There were substantial variations in NDF values between and within species (see Supplementary Table S1). The highest NDF values were recorded from *Panicum* with a maximum at 76.2%, *Paspalum notatum* at 74.9%, and *Cynodon dactylon cv Tifton* 85 at 74.7%. The minimum values for NDF were recorded for the *Pennisetum* and *Brachiaria* species, where *Pennisetum purpureum cv ILRI* 16791 and *Brachiaria cv* CIAT BR02/1752 had mean NDF values of 54.7% and 61.3%, respectively; both *Brachiaria hybrid cv Mavuno* and *Brachiaria hybrid cv Mulato II* consisted of 61.5% NDF. Mean ADF values across all tropical pasture species summarised that maximum values were measured for *Chloris gayana cv ex-Tozi* at 48.1%, *Brachiaria brizantha* at 47%, and *Panicum maximum* at 45.9%. *Cynodon dactylon*, *Cynodon nlemfuensis*, and *Brachiaria cv CIAT BRO2/1794* accounted for the lowest ADF values at 31%, 32.8%, 33.1%, respectively. Within and between species, all ADF values were below 50%, and the majority ranged between 30 and 45%, whereas the majority of NDF values ranged between 60 and 70%. Within species, *Brachiaria* had a substantial variation of ADF and NDF at 33.1–47% and 61.5–71.7%, respectively.

#### 3.2.3. Digestibility

The mean digestibility of the tropical grasses was reportedly 57.9%. Comparatively higher OMDs were recorded for *Pennisetum purpureum*, with the cultivar *Pennisetum purpureum cv Red* having the greatest absolute OMD at 69.3% across all reported tropical pasture species. Organic matter digestibility of *Brachiaria brizantha* followed that of *Pennisetum purpureum*. *Brachiaria* recorded an average of 58.1% OMD for all *Brachiaria* species and ranged between 61.9 and 54.6%. Across the genus *Brachiaria*, *Brachiaria hybrid cv Mavuno* and *Brachiaria hybrid cv Mulato II* had the greater digestibilities at 64.75% and 61.2%, respectively. The minimum average OMDs were measured for the *Chloris gayana cv ex-Tozi*, *Cynodon nlemfuensis cv Florona*, and *Cynodon dactylon cv Jiggs* varieties at 44.1%, 45%, and 49.2%, respectively. Interestingly, 73.3% of tropical pasture species from the database had OMDs that were greater than 55%, and all species were improved hybrid varieties of standard tropical pastures.

#### 3.2.4. Metabolisable Energy

Metabolisable energy of tropical pasture species in the database ranged between 5.4–9.3 MJkg^−1^ DM at a mean ME of 7.4 MJ kg^−1^ DM. Between species, the maximum absolute ME values were measured for the *Pennisetum purpureum* cultivars, consisting of a mean ME of 8.6 that ranged between 7.7 and 9.3 MJ kg^−1^ DM, followed by *Brachiaria brizantha* at a mean ME of 7.5 MJ kg^−1^ DM. The metabolisable energy level for *Brachiaria brizantha* ranged between 6.9–8.1 MJ kg^−1^ DM for *Brachiaria decumbens cv. Basilisk* and *Mulato II*, respectively. Between and within species, *Chloris gayana cv ex-Tozi* and *Cynodon nlemfuensis cv Florona* had the lowest MEs at 5.3 MJ kg^−1^ DM and 5.4 MJ kg^−1^ DM, respectively.

#### 3.2.5. Methane Gas

Estimated enteric CH_4_ emissions (g CH_4_ animal^−1^ day^−1^) of tropical pasture species are presented in Figure 4. Results did not show a substantial variation in CH_4_ emissions between and within species. Between different tropical pasture species, *Panicum maximum* at 133.19, *Paspalum notatum* at 133.15, and *Cynodon dactylon cv Tifton 85* at 133.14 g CH_4_ animal^−1^ day^−1^ had the maximum estimated enteric CH_4_ emissions. The minimum values for the estimated CH_4_ were recorded for the *Pennisetum* and *Brachiaria* species, where *Pennisetum purpureum cv ILRI 16791* and *Brachiaria cv CIAT BR02 1752* recorded mean values of 132.5 and 132.7 g CH_4_ animal^−1^ day^−1^, respectively.

### 3.3. Bioclimatic Variations in Nutritive Metrics

The nutritive values for a range of tropical pasture species are presented in Table 2, across three different bioclimatic zones (arid/dry, tropical/equatorial, and warm temperate). Greater mean DM yields were reported for the arid/dry zone, followed by the tropical/equatorial zone at 17.5 t ha^−1^ and 10.3 t ha^−1^, respectively, while DM yield in the warm temperate zone averaged only 4.0 t ha^−1^. All nutritive metrics except NDF, digestibility, ADL, and CH_4_ showed significant differences between bioclimatic zones. Significantly higher CP values were found in tropical pasture species grown in the warm temperate zone, and values were lower in warmer regions (arid and tropical). The highest mean ADF was reported for the tropical/equatorial zone followed by the arid/dry zone, whereas NDF did not significantly vary (*p* > 0.05) between zones. Nutritive values across all tropical pasture species are summarised in Supplementary Table S1, and ascending median values of main nutritive value components (CP and ME) and CH_4_ production are presented in Figure 2 and Figure 4, respectively. There were substantial variations in nutritive values of pastures within one species as well as between species in different environments.

Digestibility parameters (OMD and DMD) did not vary significantly between warm temperate and tropical zones. Ash, ADL, and OM contents were high in forage samples collected across the tropical zone, and ME was higher in pasture species grown in the arid zone. Methane production did not vary between zones. Tropical pastures grown in warm temperate climates had higher nutritive values than those from tropical/equatorial and arid/dry bioclimatic zones, although nutritive values significantly varied between the three bioclimatic zones. Overall, results showed that tropical pasture species grown in warmer regions tend to have lower nutritive values despite their higher yields compared to those in temperate regions.

### 3.4. Management and Environmental Determinants

Tropical pasture species harvested at different fixed defoliation frequencies in contrasting environments were correlated against their nutritive metrics and estimated methane production (Table 3). Results revealed that the NDF, ADF, minerals, ME, OM, and estimated CH_4_ were positively correlated with the defoliation frequency of tropical pastures. There were significant relationships for all positively correlated nutritive metrics except for OM. Crude protein, ADL, DMD, and OMD were negatively correlated with defoliation frequency. Acid detergent lignin showed no significant relationship in the data set.

According to the fitted LME model, mean annual rainfall, mean annual temperature, or mean sea level were shown to be significant only for the DM yield in the data set. Defoliation frequency and its interaction with climate were significant for all nutritive metrics (CP, NDF, ADF, ME, DMD, OMD, CH_4_, ADL, Ash) of tropical pasture species (*p* < 0.0001). There was a significant effect of defoliation intensity for NDF, ME, OMD, DMD, CH_4_, and Ash (*p* < 0.0001), whereas intensity had an interaction with climate for ADF and CP. The fitted model shows a positive effect for NDF, ADF, CH_4_, and ADL, with increasing defoliation frequencies and values of the same nutritive metrics decreasing with increasing defoliation intensity of tropical pastures (Table 4). Crude protein decreased in longer defoliation frequencies, and CP values increased with increasing defoliation intensity. Metabolisable energy and ADL values decreased due to both higher defoliation frequencies and intensities. Digestibility parameters (OMD, DMD) were negatively affected by longer defoliations, and values increased with higher intensities (Table 4).

## 4. Discussion

### 4.1. Nutritive Value of Tropical Pastures

There was a significant variation in the nutritive values of tropical pastures between and within species. According to Lean et al. [33], a minimum CP of 10–12% DM is required in dairy cattle diets in order to maintain adequate rumen function and DMI. Lactating cows require more CP (16–19%) according to their body weight, pregnancy status, level of milk production, and milk composition [33,34]. Although the present data set showed an average CP of 10.9% DM across all tropical pasture species at any given harvest interval, the CP content of hybrid cultivars (i.e., *Brachiaria*, *Pennisetum*) ranged between 16.4% and 17% DM, suggesting that hybrids only would be adequate to meet the CP requirements of lactating cows, apart from peak lactation. While the average NDF in the present data set (67.5% DM) is similar to the average value (66.2% DM) reported for tropical pastures by Van Soest [35], it is well above the minimum required NDF (25–33% DM) for lactating cows [34], and may therefore affect DMI negatively. This is especially true of NDF under longer harvesting intervals. The ADF in this data set had a greater range (previously mentioned) as well as a higher average (38.8% DM) than that reported for tropical pastures by Katoch [36] (range 31.9–35.4% DM, average 33.6% DM); furthermore, all ADF values were again above the minimum required ADF (17–21% DM) recommended for lactating cows [34]. Metabolisable energy was reported for tropical grasses as 5 to 11 MJ kg^−1^ DM [37], and the average ME of this data set was recorded as 7.5 MJ kg^−1^ DM. The present data set reported that ME was below the NRC-recommended [34] energy content required for lactating cows (8.4–10.3 MJ kg^−1^ DM, average 9.3 MJ kg^−1^ DM). Overall, the findings of this meta-analysis provide preliminary evidence about the existing limitations of tropical pastures for a productive dairy system in terms of their inherently poor average nutritive value compared with some other feed types (e.g., concentrates and temperate grasses).

An increase in the average nutritive value of tropical pastures will have a greater impact on pasture-based dairy systems. According to Ayele et al. [38] and Hall et al. [39], one of the main approaches used to increase the nutritive value and deliver quality forages on a consistent basis is to develop improved forage options and evaluate for their yield, quality, and impact on animal productivity parameters. Lowe et al. [11] observed substantial quality differences between species and between cultivars within the same species. Van Soest [35] reported that even under identical conditions, not all forages have the same quality. Results from this data set agree with Lowe et al. [11] and Van Soest [35] in that the majority of nutritive values for tropical pastures vary largely among species and within cultivars. This variation is also attributed to cultivar breeding (genotypic variation) for improved quality [9] and different physiological responses (adaptations) of individual plants to environmental factors [35]. This appeared to be more evident in this data set, as protein and digestibility values were generally higher for hybrids and cultivars within the same species (e.g., *Brachiaria*, *Pennisetum purpureum* cultivars). Apart from using hybrids and cultivars, an appropriate defoliation management regime has greater impact on average nutritive values of tropical pasture species. In addition to improving nutritive value, efforts are needed to increase and satisfy the long-term feed requirement in tropical regions. Rao et al. [40] explained the “LivestockPlus” concept for sustainable intensification of forage-based systems in the tropics under three intensification processes (genetic, ecological, and socio-economic). These enable the use of better pasture management approaches along with improved pasture species to produce better yields and nutritive values that ultimately provide livelihood (better milk production) and ecosystem (reduced GHG) benefits. Therefore, this data set that compares the nutritive values of tropical pasture species grown across different environments provides a basis for selection of quality forage options (e.g., *Brachiaria*, *Pennisetum purpureum* cultivars) that perform better across a wider geographical background, and can consequently improve tropical pasture-based dairy production systems.

### 4.2. Bioclimatic Variation

The data collected for this study covered a wide range of geographical locations (with different mean annual temperatures, mean annual rainfall values, mean sea levels, soils), and results showed interaction between defoliation management approach (defoliation frequency and intensity) and climate, through nutritive metrics. Van Soest [35] reported that climate has an effect on forage nutritive values, accounting for regional variations in composition. According to Jego et al. [41], warmer regions have been associated with taller, slow-growing, and less nutritious forages. This data set showed that the biomasses of tropical pastures harvested from arid/dry and tropical/equatorial areas were generally lower in CP, and higher in ADF, ADL, and minerals. Conversely, tropical pastures harvested from warm temperate areas were higher in CP. Both Lee et al. [27] and Lee [42] studied a range of forages in different bioclimatic zones and revealed similar results for warm and cooler areas. However, average values for NDF, ME, and digestibility metrics are different. This is explained by our data set comprising fixed defoliation frequencies for tropical pastures. These defoliation frequencies generally varied from 14 to 140 days for some tropical pastures (*Brachiaria brizantha*, *Paspalum notatum*, *Cynodon dactylon cv Tifton 85*) sampled in warm temperate areas where the frequencies ranged between 14 to 90 days in arid/dry and tropical/equatorial areas. The high NDF, low ME, and digestibility may be driven by increased structural substances (greater stem:leaf ratios) due to less intense management as well as adaptations to heat stress and water loss. Herbage production in the data set was high in warm areas, and results are in agreement with Lowe et al. [11] that DM yield is more than double in tropical areas when both the fertility and moisture are non-limiting. This greater DM yield may also explain the lower CP in warm areas that is attributed to N dilution effects caused by greater herbage accumulation [14].

The interactions of nutritive values with climate in the present data set revealed that DM yield and sward characteristics are sensitive to variables such as the environment [15,43] and the morphogenesis of plant species [44]. This may also explain the lower correlations between nutritive values across multiple pasture species and defoliation frequencies in the present data set. Therefore, the generic defoliation management options for tropical pastures are unlikely to produce better agronomic results, as they do not consider the species-specific growth nor the physiological stages induced by climate. Ruolo et al. [45] highlighted that plant-related indicators that are associated with regrowth are more sensible to use in determining pasture defoliation. This may require good information on the morphological characteristics of cultivated species. In particular, for improved tropical pastures which have not been explored to a similar degree as temperate species in order to determine the suitability for specific conditions of pasture production and management.

### 4.3. Management Determinants

The present study assessed all edible plant parts rather than focusing on their botanical compositions (leaf, stem, and dead materials). The greater range of values may be explained by the combination of different proportions of plant materials. Herbage maturity influences the forage nutritive value due to phenological and physiological changes of the plant [14]. Even though the nutritive value changes due to these phenological and physiological changes were not disentangled in our meta-analysis, the relationships reported in Table 3 explain the typical changes in nutritive value of tropical pasture species during maturity. It is widely known that CP and DMD decrease as harvesting intervals increase [46,47,48,49,50,51], and that NDF, ADF, and ADL increase with increasing harvesting intervals [14,47,48,49]. This data set is also consistent with the previous literature. In addition, the fitted LME model for the nutritive metrics explained the same relationships based on the frequency of defoliation. Defoliation intensity greatly affects the pasture nutritive value. It further explains the significant relationship between the intensity of defoliation for ME, OMD, DMD, and CH_4_, and ash. Metabolisable energy, OMD, and DMD all increased as a result of increasing defoliation intensity, while CH_4_ and ash decreased. Defoliation intensities for all tropical pastures ranged between 50 and 200 mm (100 mm average) in the data set, and this may change the vertical sward canopy structure [52] and nutritive composition [15,43] that is largely determined by the leaves and stem accumulation in each stratum [53,54,55,56,57].

### 4.4. Implications for Livestock Methane Production

Direct measurement of enteric CH_4_ has begun relatively recently due to high equipment costs and the sophisticated methodologies that are required to measure CH_4_ emissions from live animals [26]. Therefore, mathematical models are commonly used to estimate CH_4_ emissions from cattle. Current livestock models that are available to estimate CH_4_ production require many inputs that are not readily available across all experiments, and these models do not account for variations between animal breeds, regions, and climate-driven pasture nutritive values [27]. Therefore, the present meta-analysis only attempted to estimate the CH_4_ production of tropical pasture species grown in different climates as a function of NDF, based on an average DMI that is consumed by tropical dairy cattle. However, actual values may differ due to variations in DMI and diet selection, especially in grazing scenarios. The chemical composition of the forage determines the enteric CH_4_ production [58]. Forages rich in structural carbohydrates tend to result in greater CH_4_ amounts than diets higher in non-structural carbohydrates [59,60,61]. This is explained by the nutritive value results of this study that show the minimum estimated enteric CH_4_ emissions values for the *Pennisetum* and *Brachiaria* species. According to Hegarty [62] as well as Liu et al. [63], the amount of feed intake, moderated by feed digestibility and animal characteristics, affects enteric fermentation and CH_4_ production. The CH_4_ production for *Brachiaria* pasturelands was studied by Ruggieri et al. [58], who revealed that CH_4_ emissions varied from 106 to 177 g CH_4_ animal^−1^ day^−1^, at an average of 141.5 g CH_4_ animal^−1^ day^−1^. Our data set also produced similar results for a constant dry matter intake of 7.7 kg d^−1^ [28]. The Intergovernmental Panel on Climate Change (IPCC) default CH_4_ emissions factor for tropical pastures is considered to be 149 g CH_4_ animal^−1^ day^−1^ [64]; the data set presented here showed lower average values across all tropical pasture species. According to the fitted model, the effect of both intensity and frequency of defoliation, and their interaction with climate, have been shown to be significant for CH_4_ production. This relationship verified the finding of Chaves et al. [65] who found that the diet quality affected CH_4_ production. Moreover, Boadi et al. [66] demonstrated that steers that grazed on young developing growth pasture produced up to 45% less CH_4_ than if they grazed on mid- and late-season pastures. Beauchemin et al. [67] also highlighted that harvesting forage at an earlier maturity stage is a strategy that can be used to decrease enteric CH_4_ production. Meister et al. [68] also found that CH_4_ production for *Panicum maximum cv Tanzania* increases linearly with the number of grazing days, possibly reflecting a reduction in quality. De Souza Filho et al. [69] revealed that the intensity of defoliation affected CH_4_ production in the tropics, and a target height of 230–300 mm has the potential of reducing CH_4_ emissions by 13–14%. Overall, these results provide important insights for the potential of utilising lower CH_4_-producing pasture species and the potential for low emissions dairy farming through nutritive value improvements in tropical pastures through better management.

## 5. Conclusions

This meta-analysis showed that nutritive value, digestibility, and ME of tropical pastures greatly vary between and within species. Tropical pasture species *Pennisetum purpureum* followed by *Brachiaria* species showed high CP, OMD, and ME, and low NDF. Furthermore, the analysis demonstrated the variations in nutritive values of tropical pasture species across bioclimatic environments, and that nutritive values are lower in warmer and drier regions. The newer and hybrid cultivars performed better than the standard cultivars across wider bioclimatic areas, with the least quality variations suggesting their ability to deliver improved livestock forage options for tropical areas. The frequency of defoliation, defoliation intensity, climate, and their interactions had a significant effect on multiple agronomic nutritive metrics and on CH_4_ production of tropical pastures. Climate was found to be a key variable that determines tropical pasture nutritive values. This information explains the importance of setting climate-sensible defoliation strategies in order to improve tropical pasture nutritive value for sustainable dairy farming in the tropics. These results could also form the basis for further studies that research pasture agronomy from a livestock study prospective, as pastures are grown to rear livestock that ultimately produce food products for humans.

## Figures and Tables

**Table 1:** Descriptive statistics of key variables.

Item	*n*	Mean	SD	Minimum	Maximum
Climate					
MAT (°C)	294	22.8	3.45	17.5	29.30
MAR (mm)	294	1229.5	603.57	104.5	3390.0
MSL (m)	294	516.2	627.61	15.0	2393.0
Defoliation frequency (days)	294	59.51	35.68	14.0	180.0
Defoliation intensity (mm)	285	100.1	50.36	40.0	200.0
Forage yield (DM t ha^−1^)	188	7.84	6.75	0.33	46.01
Nutritive metrics (DM)					
NDF (%)	186	67.32	5.73	50.91	79.80
ADF (%)	160	38.85	5.86	24.70	57.44
ADL (%)	30	5.69	1.42	2.83	8.20
Ash (%)	88	8.75	3.18	4.40	15.20
CP (%)	254	10.97	4.08	2.11	21.10
DMD (%)	49	56.01	7.13	30.20	70.10
OMD (%)	145	57.99	7.30	33.11	72.20
ME (MJkg^−1^ DM)	136	7.41	1.16	3.50	9.75
OM (gkg^−1^)	43	894.1	81.93	590.00	957.00
CH_4_ production					
^1^ CH_4_ (g CH_4_ animal^−1^ day^−1^)	186	132.92	0.17	132.43	133.30

(*n* = number of records, MAT = mean annual temperature, MAR = mean annual rainfall, MSL = mean sea level, CP = crude protein, ADF = acid detergent fibre, NDF = neutral detergent fibre, minerals, OMD = organic matter digestibility, ME = metabolisable energy. ^1^ Methane gas production was estimated using CH_4_ (g CH_4_ animal^−1^ day^−1^) = 17.0 (±0.99) × dry matter intake + 0.03 (±0.01) × neutral detergent fibre.

**Table 2:** Least squares means (±SE) of nutritive values within bioclimatic zones described by the Köppen-Geiger climate classification system.

Nutritive Metrics	*n*	Climate			*p*-Value
		Arid/Dry	Tropical/ Equatorial	Warm Temperate	
CP (%)	245	7.15 ^b^ ± 0.97	11.25 ^a^ ± 0.42	12.02 ^a^ ± 0.76	<0.0001
NDF (%)	175	63.64 ± 2.40	67.47 ± 0.87	68.50 ± 1.21	0.1989
ADF (%)	164	37.82 ± 2.27 ^ab^	40.97 ^a^ ± 0.82	35.21 ± 1.36 ^b^	<0.0001
OMD (%)	142	-	58.76 ± 1.14	56.17 ± 1.57	0.1845
DMD (%)	53	-	54.37 ± 1.44	52.33 ± 3.60	0.6039
ME (MJ kg^−1^ DM)	142	8.67 ^a^ ± 0.55	7.60 ^b^ ± 0.18	7.19 ^b^ ± 0.25	0.0477
ADL (%)	34	4.58 ± 0.78	6.07 ± 0.42	4.50 ± 1.37	0.1913
Ash (%)	88	13.17 ^a^ ± 2.33	10.15 ^a^ ± 0.58	5.93 ^b^ ± 0.93	<0.0001
OM (g kg^−1^ DM)	47	726.17 ^b^ ± 35.30	899.35 ^a^ ± 13.57	-	<0.0001
^1^ CH_4_ (g CH_4_ animal^−1^ day^−1^)	169	132.81 ± 0.07	132.93 ± 0.03	132.96 ± 0.04	0.1858

^a,b^ Different superscript letters in the same raw data are significantly different as identified by the linear mixed models (*p* < 0.05). All nutritive metrics are given as a percentage of dry matter unless specified. *n* = number of records. Nutrient metrics are as follows: CP = crude protein, NDF = neutral detergent fibre, ADF = acid detergent fibre, OMD = organic matter digestibility, DMD = dry matter digestibility, ME = metabolisable energy, ADL = acid detergent lignin, minerals, OM = organic matter. ^1^ Methane gas production was estimated using CH_4_ (g CH_4_ animal^−1^ day^−1^) = 17.0 (±0.99) × dry matter intake + 0.03 (±0.01) × neutral detergent fibre.

**Table 3:** Pearson correlation coefficients (r) between tropical forage nutritive values and estimated methane production vs. defoliation frequency in the database.

Nutritive Metrics (% DM)	Defoliation Frequency	
	*r*	*p*-Value
NDF	0.29	<0.001
ADF	0.35	<0.001
ADL	−0.26	0.112
Minerals	0.19	0.060
CP	−0.31	<0.001
DMD	−0.36	0.005
OMD	−0.38	<0.001
ME (MJ kg^−1^ DM)	0.38	<0.001
OM (g kg^−1^)	0.14	0.334
^1^ CH_4_ (g CH_4_ animal^−1^ day^−1^)	0.29	<0.001

(Nutritive metrics are as follows: CP = crude protein, NDF = neutral detergent fibre, ADF = acid detergent fibre, OMD = organic matter digestibility, DMD = dry matter digestibility, ME = metabolisable energy, ADL = acid detergent lignin, minerals, OM = organic matter. ^1^ Methane gas production was estimated using CH_4_ (g CH_4_ animal^−1^ day^−1^) = 17.0 (±0.99) × DMI + 0.03 (±0.01) × NDF.

**Table 4:** Effect of defoliation frequency and defoliation intensity on nutritive values of tropical pasture species estimated from the linear mixed-effects models.

Nutritive Metrics	Effect	Estimate	Se	DF	t Value	*p*-Value
CP	Intercept	12.41	0.85	37	14.47	<0.0001
	Defoliation frequency	−0.082	0.007	78	−10.45	<0.0001
	Defoliation intensity	0.083	0.087	78	0.96	0.3411
NDF	Intercept	59.59	2.54	30	23.4	<0.0001
	Defoliation frequency	0.092	0.035	73	2.59	0.0117
	Defoliation intensity	0.031	0.308	73	0.1	0.9192
ADF	Intercept	39.13	3.93	39	9.95	<0.0001
	Defoliation frequency	0.038	0.011	107	3.24	0.0016
	Defoliation intensity	−0.524	0.316	107	−1.66	0.1005
ME	Intercept	6.29	0.43	40	14.55	<0.0001
	Defoliation frequency	−0.013	0.004	87	2.72	0.0078
	Defoliation intensity	0.126	0.037	87	3.37	0.0011
OMD	Intercept	50.59	2.70	40	18.71	<0.0001
	Defoliation frequency	−0.083	0.030	87	−2.72	0.0078
	Defoliation intensity	0.793	0.235	87	3.37	0.0011
DMD	Intercept	47.51	4.86	12	9.78	<0.0001
	Defoliation frequency	0.028	0.082	31	0.34	0.7336
	Defoliation intensity	0.70	0.19	31	3.59	0.0011
^1^ CH_4_	Intercept	133.18	0.12	42	1027.65	<0.0001
	Defoliation frequency	0.00031	0.00082	108	0.39	0.701
	Defoliation intensity	−0.0254	0.0099	108	−2.56	0.012
ADL	Intercept	7.77	2.56	9	3.03	0.0142
	Defoliation frequency	0.22	0.061	12	3.7	0.003
	Defoliation intensity	−0.094	0.073	12	−1.29	0.221
Minerals	Intercept	14.78	3.19	19	4.63	0.0002
	Defoliation frequency	−0.051	0.0085	59	−6.05	<0.0001
	Defoliation intensity	−0.44	0.20	59	−2.2	0.0315

(Nutritive metrics are as follows: CP = crude protein, NDF = neutral detergent fibre, ADF = acid detergent fibre, OMD= organic matter digestibility, DMD = dry matter digestibility, ME = metabolisable energy, ADL = acid detergent lignin, minerals, OM = organic matter. ^1^ Methane gas production was estimated using CH_4_ (g CH_4_ animal^−1^ day^−1^) = 17.0 (±0.99) × DMI + 0.03 (±0.01) × NDF.

## Data Availability

The data presented in this study are available in the Supplementary Materials. The digital data set of the present study is available from the corresponding author upon a reasonable request.

## Supplementary Materials

The following supporting information can be downloaded at: https://www.mdpi.com/article/10.3390/ani12141806/s1, Table S1: Nutritive value of different tropical pasture species and cultivars; Table S2: References of the studies included in the meta-analysis.
