# Supplementary material for: Comparison of Nutritive Values of Tropical Pasture Species Grown in Different Environments, and Implications for Livestock Methane Production: A Meta-Analysis
Source: Animals (Basel). 2022 Jul 14;12(14):1806. doi: 10.3390/ani12141806 (PMC9311783; doi:10.3390/ani12141806)
Supplement: Supplementary file 1 [file animals-12-01806-s001.zip › animals-1789836-supplementary.pdf]

Supplementary material

Comparison of Nutritive Values of Tropical Pasture Species Grown in Different Environments, and Implications for Livestock Methane Production: A Meta-Analysis

Priyanath Jayasinghe[1,2,*], Thiagarajah Ramilan[1], Daniel J. Donaghy[1], Keith G. Pembleton[3], David G. Barber[4]

1 School of Agriculture and Environment, Massey University, Private Bag 11 222, Palmerston North 4440, New Zealand; T.Ramilan@massey.ac.nz (R.T.); D.J.Donaghy@massey.ac.nz (D.J.D.)

2 Department of Animal Science, Faculty of Animal Science and Export Agriculture, Uva Wellassa University, Badulla, 90000, Sri Lanka.

3 Centre for Sustainable Agricultural Systems and School of Agriculture and Environmental Science, University of Southern Queensland, Toowoomba, QLD, 4350, Australia. Keith.Pembleton@usq.edu.au (K.G.P.)

4 Agri-science Queensland, Department of Agriculture and Fisheries Queensland, University of Queensland Gatton Campus, Lawes, QLD 4343, Australia. david.barber@daf.qld.gov.au (D.G.B.)

* Correspondence: P.Jayasinghe@massey.ac.nz (P.J.)

Table S1: Nutritive value (mean ± standard deviation) of different tropical pasture species and cultivars

Cultivar Name	CP	ADF	NDF	OMD	Minerals	ME	[1]CH_4
Andropogon gayanus	4.91±2.24	39.40±1.41	72.67±1.71	-	-	-	133.08±0.05
Andropogon gayanus cv Gamba	6.45±1.76	-	-	-	-	-	-
Brachiaria brizantha	11.17±3.20	47.02±6.34	66.48±7.81	-	11.49±1.33	-	132.89±0.23
Brachiaria brizantha cv Marandu	12.33±4.11	37.30±1.76	63.80±3.03	55.35±4.59	13.50±1.35	7.06±0.73	132.81±0.09
Brachiaria brizantha cv MG4	12.07±4.02	39.27±3.42	64.80±4.25	57.14±4.76	11.80±1.95	7.34±0.76	132.84±0.13
Brachiaria brizantha cv Piatá	12.53±4.82	42.27±5.84	65.57±2.98	57.00±6.35	11.77±1.46	7.32±1.02	132.87±0.09
Brachiaria brizantha cv Xaraes	11.85±2.98	38.17±2.42	65.77±1.53	55.38±5.11	11.93±1.15	7.06±0.82	132.87±0.05
Brachiaria cv CIAT BR02 / 1752	7.68±2.70	33.53±2.71	61.03±3.33	-	-	-	132.73±0.10
Brachiaria cv CIAT BRO2/1794	7.36±2.49	33.14±3.23	61.90±3.53	-	-	-	132.76±0.11
Brachiaria decumbens	10.80	41.70	71.70	-	-	-	133.05
Brachiaria decumbens cv Basilisk	11.48±5.10	41.07±2.14	67.77±3.71	54.61±6.96	11.30±2.50	6.94±1.11	132.93±0.11
Brachiaria humidicola	16.47±4.15	-	-	58.96±3.08	-	7.63±0.49	-
Brachiaria humidicola cv Llanero	12.55±4.41	40.60±0.20	67.23±1.27	56.97±5.43	11.60±0.62	7.31±0.87	132.92±0.04
Brachiaria hybrid cv Mavuno	11.00±1.41	-	61.50±2.12	64.75±2.47	-	8.56±0.40	132.75±0.06
Brachiaria hybrid cv Mulato	14.43±2.61	-	-	60.63±4.51	-	7.90±0.72	-
Brachiaria hybrid cv Mulato II	11.45±3.49	34.76±2.94	61.54±3.92	61.91±4.90	13.80±2.08	8.11±0.78	132.75±0.12
Brachiaria hybrids	11.56±4.45	45.33±4.62	64.61±3.01	57.96±7.79	8.61±2.23	7.47±1.25	132.84±0.09
Brachiaria ruziziensis	9.41±1.51	38.70	67.10	-	8.4	-	132.91
Brachiaria ruziziensis cv Kennedy	-	-	-	-	-	-	-
Cenchrus ciliaris	11.54±3.02	35.97±0.67	67.07±1.46	-	-	-	132.91±0.04
Cenchrus ciliaris cv Bilocla	6.10±2.96	-	-	-	-	-	-
Cenchrus ciliaris cv Gayndah	6.1±3.39	-	-	-	-	-	-
Chloris gayana cv ex-Tozi	7.00±2.65	48.10±2.33	72.87±0.81	44.56±10.23	8.80±0.62	5.33±1.64	133.09±0.02
Chloris gayana cv Callide	12.53±0.60	-	65.73±0.47	-	-	-	-
Chloris gayana cv. Katambora	7.25±4.17	-	-	-	-	-	-
Cynodon dactylon	10.86±5.86	31.00±3.17	70.34±7.54	57.50±2.52	5.92±1.50	7.40±0.40	133.01±0.23
Cynodon dactylon cv Jiggs	11.04±5.30	33.84±3.69	67.61±5.10	49.25±6.01	6.24±1.91	6.08±0.96	132.93±0.15

Cynodon dactylon cv Russell	10.82±5.59	33.38±2.94	67.90±2.56	-	5.88±1.10	-	132.94±0.08
Cynodon dactylon cv Tifton 85	14.83±3.15	44.50±2.55	74.73±4.16	50.72±6.05	-	6.31±0.97	133.14±0.12
Cynodon dactylon cv Tifton 9	10.02±4.53	35.46±2.01	66.10±2.43	-	5.72±0.84	-	132.88±0.07
Cynodon nlemfuensis	-	32.84±1.14	74.20±1.46	54.68±2.31	-	6.95±0.37	133.13±0.04
Cynodon nlemfuensis cv Florona	-	-	-	45.05±3.82	-	5.41±0.61	-
Eragrostis curvula	6.95±3.74	-	-	-	-	-	-
Hemarthria altissima cv Floralta	-	-	-	50.68±4.95	-	6.31±0.79	-
Imperata cylindrica	10.67±1.05	-	-	-	-	-	-
Panicum coloratum cv Bambatsi	7.05±2.86	-	-	-	-	-	-
Panicum maximum	9.28±2.48	45.93±3.41	76.26±3.30	62.47±3.06	7.05±1.56	8.20±0.49	133.19±0.10
Panicum maximum cv Mombosa	7.12±3.23	42.30±2.80	68.63±2.92	-	-	-	132.96±0.09
Panicum maximum cv Tanzania	7.13±1.13	40.80±1.88	68.73±1.46	-	-	-	132.96±0.04
Paspalum atratum	5.60±1.37	38.73±1.61	66.56±1.87	-	-	-	-
Paspalum notatum	-	35.48±1.32	74.92±0.68	51.92±2.22	-	6.51±0.35	133.15±0.02
Paspalum notatum cv Argentina	9.84±5.41	34.16±2.27	65.24±3.27	-	6.12±1.08	-	132.86±0.10
Paspalum notatum cv Pensacola	9.82±4.37	35.66±1.76	67.02±2.35	51.80±2.02	5.68±0.94	6.49±0.32	132.91±0.07
Pennisetum purpureum	10.79±3.22	42.97±4.71	67.99±7.27	59.70±6.38	9.12±3.61	7.75±1.02	132.94±0.22
Pennisetum purpureum cv 3rd generation	16.27±1.75	-	-	65.44±1.33	-	8.67±0.21	-
Pennisetum purpureum cv Dwarf	11.46±1.06	-	-	-	-	-	-
Pennisetum purpureum cv ILRI 16791	10.23±2.07	38.93±2.61	54.70±0.10	63.23±3.67	13.17±0.78	8.32±0.59	132.54±0.00
Pennisetum purpureum cv India	16.35±2.01	-	-	65.84±0.22	-	8.74±0.04	-
Pennisetum purpureum cv King	10.46±2.01	42.42±4.05	70.93±5.20	-	-	-	133.03±0.16
Pennisetum purpureum cv Kobe	17.00±2.45	-	-	67.34±0.75	-	8.98±0.12	-
Pennisetum purpureum cv Mott	13.83±1.64	36.08±2.37	63.36±1.97	67.18±4.22	-	8.95±0.68	132.80±0.06
Pennisetum purpureum cv NB 21	15.75±0.52	-	-	-	-	-	-
Pennisetum purpureum cv Red	15.25±2.57	-	-	69.34±2.61	-	9.30±0.42	-
Pennisetum purpureum cv Taiwan	15.60±2.31	-	-	66.19±0.35	-	8.79±0.06	-
Pennisetum purpureum cv TaiwanA25	10.60±1.83	-	-	-	-	-	-
Pennisetum purpureum cv Zanzibar	16.70±3.86	-	-	66.44±2.30	-	8.83±0.37	-

Urochloa mosambicensis	7.80±2.96	-	-	-	-	-	-

(Nutrient metrics are CP = crude protein, NDF = neutral detergent fibre ADF = acid detergent fibre, OMD= organic matter digestibility, DMD = dry matter digestibility, ME = metabolisable energy , ADL = acid detergent lignin, minerals, , OM = organic matter. [1]methane gas production estimated using CH_4 (g CH_4 animal^{-1} day^{-1}) = 17.0 (±0.99) × DMI + 0.03 (±0.01) × NDF.

Table S2: References of the studies included in the meta-analysis.

Reference	Country	Climate
[1]	Thailand	Tropical/ equatorial
[2]	Thailand	Tropical/ equatorial
[3]	Ethiopia	Arid/ dry
[4]	Ghana	Tropical/ equatorial
[5]	Thailand	Tropical/ equatorial
[6]	Philippines	Tropical/ equatorial
[7]	Ethiopia	Warm temperate
[8]	USA	Warm temperate
[9]	Ethiopia	Tropical/ equatorial
[10]	Brazil	Arid/ dry
[11]	USA	Warm temperate
[12]	Malaysia	Tropical/ equatorial
[13]	Vietnam	Tropical/ equatorial
[14]	Brazil	Arid/ dry
[15]	Brazil	Arid/ dry
[16]	Ghana	Tropical/ equatorial
[17]	Brazil	Tropical/ equatorial
[18]	Brazil	Warm temperate
[19]	Rwanda	Tropical/ equatorial
[20]	Venezuela	Tropical/ equatorial
[21]	USA	Warm temperate
[22]	USA	Warm temperate
[23]	Cameroon	Tropical/ equatorial
[24]	Vietnam	Tropical/ equatorial
[25]	USA	Warm temperate
[26]	USA	Warm temperate
[27]	Australia	Warm temperate
[28]	Thailand	Tropical/ equatorial
[29]	Thailand	Tropical/ equatorial
[30]	Mozambique	Arid/ dry
[31]	Sri Lanka	Tropical/ equatorial
[32]	Brazil	Tropical/ equatorial
[33]	Kenya	Tropical/ equatorial
[34]	Kenya	Warm temperate
[35]	Kenya	Tropical/ equatorial

1. Hare, M.D. Effect of cutting interval on yield and quality of two *Panicum maximum* cultivars in Thailand. *Trop. Grassl.-Forrajes Trop.* **2013**, *1*, 87-89.
2. Hare, M.D. Effect of cutting interval on yield and quality of three *brachiaria* hybrids in Thailand. *Trop. Grassl.-Forrajes Trop.* **2013**, *1*, 84-86.
3. Tessema, Z.K.; Mihret, J.; Solomon, M. Effect of defoliation frequency and cutting height on growth, dry-matter yield and nutritive value of Napier grass (*Pennisetum purpureum* (L.) Schumach). *Grass Forage Sci.* **2010**, *65*, 421-430, doi:10.1111/j.1365-2494.2010.00761.x.

4. Ansah, T. Herbage yield and chemical composition of four varieties of Napier (*Pennisetum purpureum*) grass harvested at three different days after planting. *Agr. Biol. J. N. Am.* **2010**, *1*, 923-929.
5. Lounglawan, P.; Lounglawan, W.; Suksombat, W. Effect of Cutting Interval and Cutting Height on Yield and Chemical Composition of King Napier Grass (*Pennisetum Purpureum x Pennisetum Americanum*). *APCBEE Procedia* **2014**, *8*, 27-31, doi:https://doi.org/10.1016/j.apcbee.2014.01.075.
6. Bacorro, T.J. Herbage dry matter yield, nutrient composition and in vitro gas production of mulato II and mombasa grasses at 30- and 45- day cutting intervals. *Philipp. J. Vet. Anim. Sci.* **2018**, *44*, 86-89.
7. Wassie, W.A.; Tsegay, B.A.; Wolde, A.T.; Limeneh, B.A. Evaluation of morphological characteristics, yield and nutritive value of *Brachiaria* grass ecotypes in northwestern Ethiopia. *Agric. Food Secur.* **2018**, *7*, 89, doi:10.1186/s40066-018-0239-4.
8. Johnson, C.R.; Reiling, B.A.; Mislevy, P.; Hall, M.B. Effects of nitrogen fertilization and harvest date on yield, digestibility, fiber, and protein fractions of tropical grasses. *J. Anim. Sci.* **2001**, *79*, 2439-2448, doi:10.2527/2001.7992439x.
9. Bayble, T, Melaku, S.; Prasad, N.K. Effects of cutting dates on nutritive value of Napier (*Pennisetum purpureum*) grass planted sole and in association with Desmodium (*Desmodium intortum*) or Lablab (*Lablab purpureus*). *Livest. Res. Rural. Dev.* **2007**, *19*.
10. Ribeiro Jr, G.O.; Teixeira, A.M.; Velasco, F.O.; Faria Júnior, W.G.; Pereira, L.G.R.; Chaves, A.V.; Gonçalves, L.C.; McAllister, T.A. Production, Nutritional Quality and In vitro Methane Production from *Andropogon gayanus* Grass Harvested at Different Maturities and Preserved as Hay or Silage. *Asian-Australas. J. Anim. Sci.* **2014**, *27*, 330-341, doi:10.5713/ajas.2013.13161.
11. Arthington, J.D.; Brown, W.F. Estimation of feeding value of four tropical forage species at two stages of maturity1,2. *J. Anim. Sci.* **2005**, *83*, 1726-1731, doi:10.2527/2005.8371726x.
12. Haryani, H.; Norlindawati, A.P.; Norfadzrin, F.; Aswanimiyuni, A.; Azman, A. Yield and nutritive values of six napier (*Pennisetum purpureum*) cultivars at different cutting age. *Malays. J. Vet. Res.* **2018**, *9*, 6-12.
13. Van Man, N.; Wiktorsson, H. Forage yield, nutritive value, feed intake and digestibility of three grass species as affected by harvest frequency. *Trop. Grassl.-Forrajes Trop.* **2003**, *37*, 101-110.
14. Kozloski, G.V.; Perottoni, J.; Ciocca, M.L.S.; Rocha, J.B.T.; Raiser, A.G.; Sanchez, L.M.B. Potential nutritional assessment of dwarf elephant grass (*Pennisetum purpureum Schum. cv. Mott*) by chemical composition, digestion and net portal flux of oxygen in cattle. *Anim. Feed Sci. Technol.* **2003**, *104*, 29-40, doi:https://doi.org/10.1016/S0377-8401(02)00328-0.
15. Kozloski, G.V.; Perottoni, J.; Sanchez, L.M.B. Influence of regrowth age on the nutritive value of dwarf elephant grass hay (*Pennisetum purpureum Schum. cv. Mott*) consumed by lambs. *Anim. Feed Sci. Technol.* **2005**, *119*, 1-11, doi:https://doi.org/10.1016/j.anifeedsci.2004.12.012.
16. Meale, S.J.; Chaves, A.V.; Baah, J.; McAllister, T.A. Methane Production of Different Forages in In vitro Ruminal Fermentation. *Asian-Australas. J. Anim. Sci.* **2012**, *25*, 86-91, doi:10.5713/ajas.2011.11249.
17. Lima, D.M., Filho, A.L.A.; Lima, P.M. T.; Sakita, G. Z.; Dias e Silva, T. P.; McManus, C.; Abdalla, A. L.; Louvandini, H. Morphological characteristics, nutritive quality, and methane production of tropical grasses in Brazil. *Pesqui. Agropecu. Bras.* **2018**, *53*, 323-331.
18. Taffarel, L.E.; Mesquita, E.E.; Castagnara, D.D.; Galbeiro, S.; Costa, P.B.; de Oliveira, P.S.R. Tifton 85 grass responses to different nitrogen levels and cutting intervals. *Semina: Ciências Agrárias.* **2016**, *37*, 2067-2083.
19. Mutimura, M.; Cyprian, E.; Rao, I. M.; Nsahlai, I. V. Effect of cutting time on agronomic and nutritional characteristics of nine commercial cultivars of *Brachiaria* grass compared with Napier grass during establishment under semi-arid conditions in Rwanda . *Afr. J. Agric. Res.* **2017**, *12*, 2692-2703, doi:https://doi.org/10.5897/AJAR2017.12474.
20. González. R.; M.; Fondevila, M.; Barrios Urdaneta, A.; Newman, Y. In vitro gas production from buffel grass (*Cenchrus ciliaris L.*) fermentation in relation to the cutting interval, the level

of nitrogen fertilisation and the season of growth. *Anim. Feed Sci. Technol.* **1998**, *72*, 19-32, doi:https://doi.org/10.1016/S0377-8401(97)00181-8.
21. Vendramini, J.M.B.; Sollenberger, L.E.; Soares, A.B.; Da Silva, W.L.; Sanchez, J.M.; Valente, A.L.; Aguiar, A.D.; Mullenix, M.K. Harvest frequency affects herbage accumulation and nutritive value of *brachiaria* grass hybrids in Florida. *Trop. Grassl.-Forrajes Trop.* **2014**, *2*, 197-206, doi:10.17138/tgft(2)197-206.
22. Inyang, U.; Vendramini, J.M.B.; Sellers, B.; Silveira, M.L.A.; Lunpha, A.; Sollenberger, L.E.; Adesogan, A.; Paiva, L.M. Harvest frequency and stubble height affect herbage accumulation, nutritive value, and persistence of 'Mulato II' *Brachiariagrass*. *Forage and Grazinglands* **2010**, *8*, doi:10.1094/fg-2010-0923-01-rs.
23. Enoh, M. B.; Kijora, C.; Peters, K. J.; Yonkeu, S. Effect of stage of harvest on DM yield, nutrient content, in vitro and in situ parameters and their relationship of native and *Brachiaria* grasses in the Adamawa Plateau of Cameroon. *Livest. Res. Rural. Dev.* **2005**, *17*.
24. McRoberts, K.C.; Parsons, D.; Ketterings, Q.M.; Hai, T.T.; Quan, N.H.; Ba, N.X.; Nicholson, C.F.; Cherney, D.J.R. Urea and composted cattle manure affect forage yield and nutritive value in sandy soils of south-central Vietnam. *Grass Forage Sci.* **2018**, *73*, 132-145, doi:10.1111/gfs.12289.
25. Dore, R.T. Comparing bermudagrass and bahiagrass cultivars at different stages of harvest for dry matter yield and nutrient content. Master of Science (MS), Louisiana State University and Agricultural and Mechanical College, USA, 2006.
26. Siqueira da Silva, H.M.; Vendramini, J.M.B.; Leite de Oliveira, F.C.; Soares Filho, C.V.; Kaneko, M.; Silveira, M.L.; Dalmazo Sanchez, J.M.; Yarborough, J.K. Harvest frequency effects on herbage characteristics of 'Mavuno' *brachiaria*grass. *Crop Sci.* **2020**, *60*, 1113-1122, doi:https://doi.org/10.1002/csc2.20046.
27. Ehrlich, W.; Cowan, R.; Lowe, K. Managing rhodes grass (*Chloris gayana*) cv. Callide to improve diet quality. 1. Effects of age of regrowth, strip grazing and mulching. *Trop. Grassl.-Forrajes Trop.* **2003**, *37*, 33-44.
28. Tudsri, S.; Ishii, Y.; Numaguchi, H.; Prasanpanich, S. The effect of cutting interval on the growth of *Leucaena leucocephala* and three associated grasses in Thailand. *Trop. Grassl.-Forrajes Trop.* **2002**, *36*, 90-96.
29. Hare, M.; Saengkham, M.; Kaewkunya, C.; Tudsri, S.; Suriyajantratong, W.; Thummasaeng, K.; Wongpichet, K. Effect of cutting on yield and quality of *Paspalum atratum* in Thailand. *Trop. Grassl.-Forrajes Trop.* **2001**, *35*, 144-150.
30. Muir, J.; Abrao, L. Agronomic evaluation of 10 cultivated grasses in semi-arid Mozambique. *Trop. Grassl.-Forrajes Trop.* **1999**, *33*, 34-39.
31. Premarathne, S. Effect of Frequancy of cutting on yeild and chemical composition of four tropical grassess. *J. Natl. Sci. Found. Sri Lanka* **1993**, *21*, 189-194.
32. Pequeno, D.N.L.; Pedreira, C.G.S.; Sollenberger, L.E.; de Faria, A.F.G.; Silva, L.S. Forage accumulation and nutritive value of *Brachiaria*grasses and Tifton 85 bermudagrass as affected by harvest frequency and irrigation. *Agron. J.* **2015**, *107*, 1741-1749, doi:10.2134/agronj15.0115.
33. Ondiko, C. N.; Njunie M. N.; Njarui D. M. G.; Auma E.; Ngode L. Effects of cutting frequency on forage production and nutritive value of *Brachiaria* grass cultivars in coastal lowlands of Kenya, Climate smart *Brachiaria* grasses for improving livestock production in East Africa: Kenya Experience: Proceedings of a workshop, Naivasha, Kenya, 14-15, Eds. Njarui, D.M.; Gichangi, E.M.; Ghimire, S.R.; Muinga, R.W.; Kenya Agricultural and Livestock Research Organization: Nairobi, Kenya, **2016**.
34. Nyambati, E. M.; Ayako, W.; Chelimo, E. J.; Njarui, D. M. G. Production and nutritive quality of *Brachiaria* grass cultivars subjected to different cutting intervals in the cool sub-humid highlands of central Kenya, Climate smart *Brachiaria* grasses for improving livestock production in East Africa: Kenya Experience: Proceedings of a workshop, Naivasha, Kenya, 14-15, Eds. Njarui, D.M.; Gichangi, E.M.; Ghimire, S.R.; Muinga, R.W.; Kenya Agricultural and Livestock Research Organization: Nairobi, Kenya, **2016**.

35. Njarui, D. M. G.; Gatheru, M.; Ghimire, S. R.; Mureithi, J. G. Effects of seasons and cutting intervals on productivity and nutritive value of *Brachiaria* grass cultivars in semi-arid eastern Kenya, Climate smart *Brachiaria* grasses for improving livestock production in East Africa: Kenya Experience: Proceedings of a workshop, Naivasha, Kenya, 14-15, Eds. Njarui, D.M.; Gichangi, E.M.; Ghimire, S.R.; Muinga, R.W.; Kenya Agricultural and Livestock Research Organization: Nairobi, Kenya, **2016**.
